# Immune Checkpoints Contribute Corneal Immune Privilege: Implications for Dry Eye Associated with Checkpoint Inhibitors

**DOI:** 10.3390/ijms21113962

**Published:** 2020-05-31

**Authors:** Junko Hori, Tomoyuki Kunishige, Yuji Nakano

**Affiliations:** 1 Department of Ophthalmology, Nippon Medical School, 1-1-5 Sendagi, Bunkyo-ku, Tokyo 113-8603, Japan; s9038@nms.ac.jp (T.K.); n-yuji@nms.ac.jp (Y.N.); 2 Department of Ophthalmology, Nippon Medical School, Tama-Nagayama Hospital, 1-7-1 Nagayama, Tama, Tokyo 206-8512, Japan

**Keywords:** immune privilege, immune checkpoints, immune-related adverse events, anterior chamber-associated immune deviation, regulatory T cells, corneal transplantation, dry eye, programmed death-1, programmed death ligand-1, V-domain Ig suppressor of T cell activation

## Abstract

The eye is provided with immune protection against pathogens in a manner that greatly reduces the threat of inflammation-induced vision loss. Immune-mediated inflammation and allograft rejection are greatly reduced in the eye, a phenomenon called ‘immune privilege’. Corneal tissue has inherent immune privilege properties with underlying three mechanisms: (1) anatomical, cellular, and molecular barriers in the cornea; (2) an immunosuppressive microenvironment; and (3) tolerance related to regulatory T cells and anterior chamber-associated immune deviation. This review describes the molecular mechanisms of the immunosuppressive microenvironment and regulatory T cells in the cornea that have been elucidated from animal models of ocular inflammation, especially those involving corneal transplantation, it also provides an update on immune checkpoint molecules in corneal and systemic immune regulation, and its relevance for dry eye associated with checkpoint inhibitor therapy.

## 1. Introduction

In the eye, corneal endothelium and neural retina are unable to proliferate in vivo. Damage to the ocular tissues from excessive inflammatory reactions may lead to loss of sight. The eye, like the brain and reproductive organs, has developed inherent immune privilege and inflammation self-regulated in order to preserve the eye’s function [1,2,3].

The ocular immune privilege was discovered by Medawar in the 1940s [4]. Medawar studied the fate of allogenic skin graft in the anterior chamber of the eye and discovered that skin allografts placed in the anterior chamber survived for prolonged periods of time, whereas skin allografts placed at conventional body sites were rejected [4]. Through this observation, he determined that the anterior chamber of the eye had the properties of an immune-privileged site. Billingham, a colleague of Medawar, provided evidence that the cornea functions as an immune-privileged tissue [5]. Ocular immune privilege provides the cornea with immune protection from immunogenic inflammation that may lead to distortions of the visual axis and blindness [6,7,8,9]. Streilein and colleagues found that this was not just due to immunological ignorance, but also active immunosuppressive mechanisms [1,2,3]. Active mechanisms to achieve corneal immune privilege consist of two strategies: (1) development of an immunosuppressive microenvironment in the anterior segment of the eye, and (2) tolerance related to regulatory T cells and anterior chamber-associated immune deviation.

This review provides an updated overview of the molecular mechanisms of the immunosuppressive microenvironment and regulatory T cells that is mediated immune checkpoints molecules expressed in the cornea. Various immune checkpoint molecules are constitutively expressed in the cornea, and they regulate immune responses to prevent inflammation-mediated corneal tissue damage. Meanwhile, immune checkpoint inhibitors have become new therapies for cancer. In clinical settings, administration of immune checkpoint inhibitors enhances the immune system and causes immune-related adverse events (irAEs) including dry eye. Role of immune checkpoints on regulation of dry eye has not been studied and unclear, so far. This review also provides implications for underlying mechanisms of dry eye associated with immune checkpoint inhibitors.

## 2. Immune Suppressive Microenvironment in the Anterior Segment of the Eye

In response to threats to vision, the anterior segment of the eye has soluble and cell surface immunomodulatory factors that act within the eye to suppress cells and molecules that mediate inflammation. This intraocular milieu is called the immune-suppressive microenvironment. The functions of the various cells and factors that manage immune responses in the eye are shown in Table 1 [1,2,3,10,11,12,13,14,15,16,17,18,19,20,21]. Among those factors, α-melanocyte-stimulating hormone (α-MSH), vasoactive intestinal peptide (VIP), calcitonin gene-related peptide (CGRP), TGF-β2, and TSP-1 regulate the functions of macrophages and cDCs. TGF-β2 and TSP-1 are essential factors for the induction of ACAID. ACAID is a form of systemic tolerance to antigens placed in the anterior chamber of the eye [22,23]. Injection of antigen material into the anterior chamber generates a systemic immune response that consists of primed, clonally expanded cytotoxic T cell precursors and B cells secreting large concentrations of IgG_1_, a non-complement-fixing antibody, whereas ACAID prevents the development and expression of DTH responses in an antigen-specific manner through the down-regulation of CD4^+^ Th1 and Th2 cells [24,25,26,27]. As shown in Table 1, various immunomodulatory factors are expressed in corneal endothelial cells and in the iris–ciliary body.

**Figure 1:**
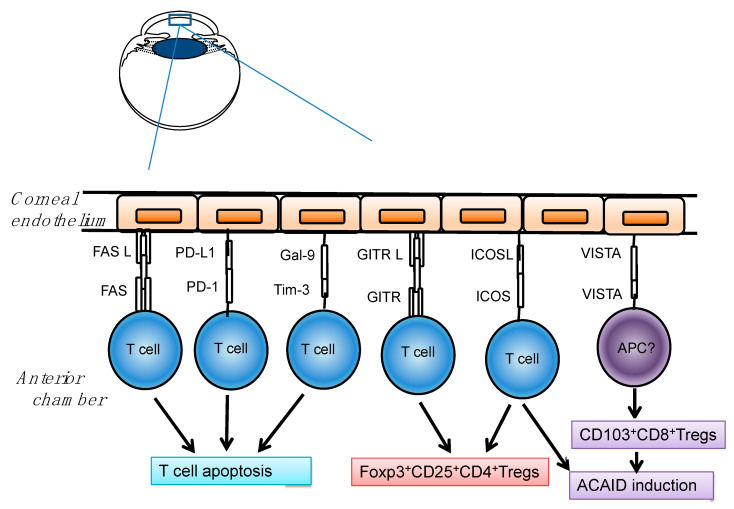
Constitutive expression of immune checkpoints in endothelial cells of the cornea. Programmed death (PD)-L1 induces apoptosis of PD-1+ T cells. Fas Ligand (FasL) induces T-cell apoptosis via Fas. Galectin-9 (Gal-9) also induces apoptosis of T cells and protects corneal endothelium. Glucocorticoid-induced tumor necrosis factor receptor family-related protein (GITR)- ligand (GITRL) functions to induce Foxp3^+^CD25^+^CD4^+^ Tregs via GITR. Inducible costimulatory molecule (ICOS)-ligand (ICOSL) also induces Foxp3^+^CD25^+^CD4^+^ Tregs via ICOS. V-domain Ig suppressor of T cell activation (VISTA) is involved in induction of CD103^+^CD8^+^ Tregs and anterior chamber-associated immune deviation (ACAID). Reprinted from Prog Retin Eye Res. 72, 100758. Hori, J.; Yamaguchi, T.; Keino, H.; Hamrah, P.; Maruyama, K. Immune privilege in corneal transplantation. Copyright (2019), with permission from Elsevier [3].

We have revealed that inhibitory costimulatory signaling molecules such as programmed death ligand-1 (PD-L1, B7-H1) [28], inducible costimulatory molecule ligand (ICOSL, B7-H2, B7-related protein (B7RP)-1) [29], V-domain Ig suppressor of T cell activation (VISTA, PD-1H) [30], glucocorticoid-induced tumor necrosis factor (TNF) receptor family-related protein ligand (GITRL) [31], and galectin-9 [32], are constitutively expressed in the corneal tissue and involved in immune suppression in the cornea (Figure 1). These inhibitory costimulatory signal molecules play an important role in the regulation of antigen-specific T-cell-mediated immune responses. The magnitude of the immune response is regulated by a balance between co-stimulatory and inhibitory signals. Inhibitory co-stimulatory molecules are referred to as “immune checkpoints” and are necessary for maintaining immune homeostasis and preventing inflammatory tissue damage.

## 3. Role of Immune Checkpoints Molecules in the Cornea

### 3.1. Immune Checkpoints-Mediated T Cell Apoptosis in the Cornea

#### 3.1.1. Fas Ligand and Fas

Fas Ligand (FasL) and Fas are members of the tumor necrosis factor (TNF)-receptor and TNF family, respectively. The ligation of Fas with FasL results in the activation of a caspase cascade that initiates apoptosis [33,34,35,36,37]. Corneal cells, especially endothelium and epithelium, constitutively express FasL, a molecule that functions as a receptor for Fas [11]. Fas is expressed on many cells but particularly on activated T lymphocytes. Engagement of Fas on T cells by FasL triggers apoptosis among the Fas-bearing cells, and this mechanism of deletion has been implicated in the ability of orthotopic and heterotopic corneal allografts to resist immune rejection [11,16,21,38,39]. Constitutive expression of FasL on corneal endothelium is critical to its immune-privileged status. FasL renders corneal endothelium resistant to immune destruction (immune efferent phase). This is revealed by the persistence of endothelial cells in allografts at heterotopic sites of pre-sensitized mice, where stroma but not endothelium is being destroyed [39]. Moreover, some evidence indicates an immunomodulatory role for FasL in the induction of alloimmunity (immune afferent phase) [39]. Stroma–endothelial allografts only induced systemic donor-specific DH (and their rejection) if the grafts were derived from FasL-deficient (B6Smn.C3H-gld) donor [38]. Thus, FasL, perhaps by triggering apoptosis in naïve, alloreactive, donor-specific T cells, prevents allosensitization.

#### 3.1.2. PD-Ligand 1 and PD-1

Programmed death (PD)-1 is a negative regulatory molecule, which is a member of the B7-CD28 superfamily [40]. This molecule is a type I transmembrane protein that was originally identified in a T cell line undergoing programmed cell death. It has been found to be expressed on activated T and B cells and on a subset of thymocytes [40]. PD-1 contains an ITIM in its cytoplasmic tail [41,42] that negatively regulates T cell Ag receptor signaling through interactions with specific ligands. PD-L1 (B7-H1) is a B7 family molecule that binds to programmed death (PD)-1 on the surface of activated cells and sends inhibitory signals to the T cells [43]. In ocular tissues, PD-L1 is constitutively expressed on endothelial cells of the cornea, some stromal cells, iris–ciliary body, and the neural retina [28]. The rejection reaction after corneal transplantation is enhanced by blockade of PD-L1 or PD-1 with antibodies [28]. PD-L1 expressed in the cornea induces apoptosis of PD-1-expressing T cells, and this deletion of effector T cells in the cornea results in inhibition of the effector phase of the rejection reaction [28]. Interestingly, the T-cell apoptosis mediated by PD-L1 has only been observed in immune-privileged tissues or sites such as tumors, liver, and cornea [44,45]. It remains unclear whether or not PD-L1 and FasL play a nonredundant or cooperative role to delete effector T cells in the eye.

**Figure 2:**
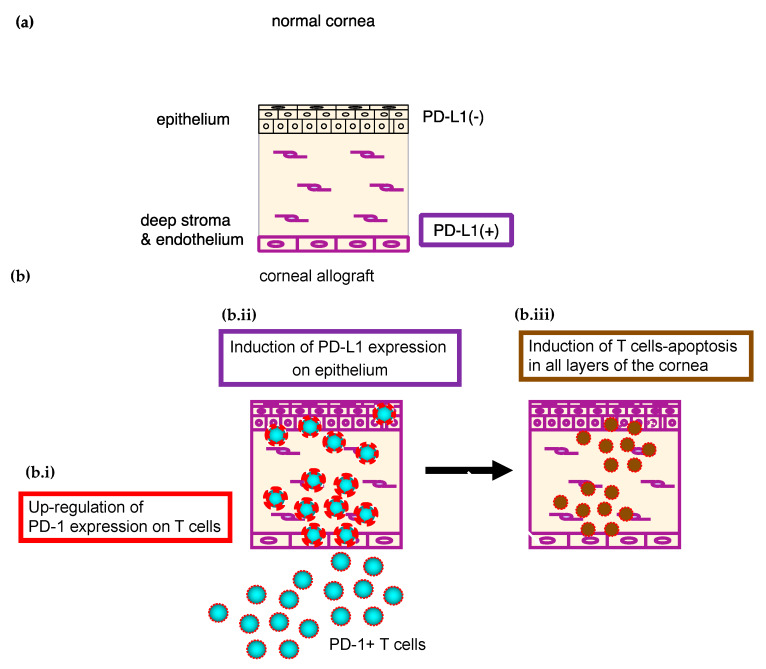
PD-L1/PD-1-mediated T-cell apoptosis in the cornea. (**a**) PD-L1 is constitutively expressed in endothelial cells and some stromal cells, but not in epithelial cells. (**b**): (b.i) PD-1 expression on the surface of T cells is up-regulated after contact with the corneal cells. (b.ii) PD-L1 expression on epithelial cells is induced in the presence of IFN-γ. (b.iii) T-cell apoptosis mediated by PD-L1 and PD-1 is thus induced in each of the layers (epithelium, stroma and endothelium) of the cornea. Reprinted from Prog Retin Eye Res. 72, 100758. Hori, J.; Yamaguchi, T.; Keino, H.; Hamrah, P.; Maruyama, K. Immune privilege in corneal transplantation. Copyright (2019), with permission from Elsevier [3].

In vitro culture system of corneal tissue and T cells, PD-L1 expressed in the cornea shows local immunosuppressive activity and protects corneal endothelial cells from killing by T cells [28]. This system permits complete elimination of any involvement of the secondary lymphatic organs and thus allows isolation and analysis of only the effector phase of the rejection reaction when the corneal endothelial cells have been destroyed by effector T cells. In vitro, PD-L1 expressed in the corneal cells not only inhibited corneal endothelial destruction by allo-reactive T cells, but also inhibited bystander damage caused by activated T cells that are specific to third-party antigen. Moreover, PD-1 on the surface of the T cells was up-regulated as a result of contact with the corneal cells, thus accelerating apoptosis (Figure 2). Moreover, PD-L1 expression on epithelial cells was induced in the presence of inflammatory cytokines such as IFN-γ (Figure 2). T-cell apoptosis mediated by PD-L1 and PD-1 is thus induced in all three (epithelium, stroma and endothelium) layers of the cornea (Figure 2).

As described above, the PD-L1/PD-1 pathway is especially involved in interactions between the effector T cells and corneal cells within the eye rather than in the immune responses in the secondary lymphatic organs [28]. These molecules thus contribute to the maintenance of the local immune-suppressive microenvironment in the eye.

#### 3.1.3. Galectin-9 and Tim-3

The T-cell immunoglobulin and mucin domain (Tim) family is a novel group of immune checkpoint molecules with a conserved structure and important immunologic functions, including T-cell activation, induction of T-cell apoptosis, T-cell tolerance, and the clearance of apoptotic cells [46,47,48]. Tim-3 is a member of the Tim family specifically expressed on murine T helper (Th)1 cells, but not on Th2 cells [49]. Expression of Tim-3 is detectable only after several rounds of stimulation on CD4 and CD8 cells under Th1 conditions [50,51]. Tim-3 is also expressed constitutively on macrophages and dendritic cells, and serves opposing roles in the innate and adaptive immune systems [52]. Galectin-9 (Gal-9) has recently been identified as a Tim-3 ligand that negatively regulates Th1 immunity by inducing cell death in effector Th1 cells [53].

Gal-9 is constitutively expressed on the corneal epithelium, endothelium and iris–ciliary body in normal mouse eyes [32]. Allograft survival in recipients treated with anti-Tim-3 antibody (mAb) or anti-Gal-9 mAb was significantly less than that in control recipients [32]. In vitro culture system of corneal tissue and T cells, destruction of corneal endothelial cells by allo-reactive T cells was enhanced when the cornea was pretreated with anti-Gal-9 mAb. Apoptosis of CD4^+^ T cells was significantly suppressed after the co-culture of allo-reactive T cell with Gal-9-blocked cornea, compared to Gal-9-expressing cornea [32]. Gal-9 expressed on corneal endothelial cells thus protects these cells from destruction by allo-reactive T cells within the cornea.

### 3.2. Immune Checkpoints-Mediated Treg and Peripheral Tolerance in the Cornea

#### 3.2.1. ICOS Ligand and ICOS

Inducible co-stimulator (ICOS) is a third member of CD28/CTLA4 family of molecules [54], and it is expressed on activated T cells and resting memory T cells [54,55,56]. ICOS ligand (ICOSL), which has homology to B7 molecules and is called B7-related protein-1 (B7RP-1, B7-H2,), is expressed on B cells and macrophages [55,57]. ICOS/ICOSL (B7-H2, B7RP-1) signals have been implicated in the differentiation and function of T cells. In the eye, ICOSL mRNA is constitutively expressed in the cornea, iris–ciliary body and retina [29]. Corneal allograft survival in ICOS^−/−^ recipients and wild-type (WT) recipients treated with anti-ICOSL mAb was significantly shorter than in control recipients. In addition, ACAID was induced less efficiently in ICOS^−/−^ mice. In vitro culture system of corneal tissue and T cells, the destruction of corneal endothelial cells by allo-reactive ICOS^−/−^ T cells was enhanced compared with WT T cells. After co-incubation with allogeneic corneal tissue, the proportion of Foxp3^+^CD4^+^ Tregs was significantly greater among WT T cells than among ICOS^−/−^ T cells. Thus, expression of ICOSL in the cornea and the ICOS-mediated induction of Foxp3^+^CD4^+^ Tregs contribute to successful corneal allograft survival [29].

#### 3.2.2. VISTA(PD-1H)

V-domain Ig suppressor of T cell activation (VISTA)/PD-1H is a novel co-inhibitory immune checkpoint receptor and ligand whose extracellular domain bears homology to the B7 family ligand PD-L1 [58,59,60]. VISTA is primarily expressed on hematopoietic cells, and VISTA expression is highly regulated on myeloid antigen-presenting cells (APCs) and T cells [58]. VISTA can function as a receptor as well as a ligand [61]. Indeed, structural modeling suggests homology to PD-1 [59] and PD-L1 [58]. Soluble VISTA-Ig fusion protein or VISTA expression on APCs inhibits T cell proliferation and cytokine production in vitro [58].

**Figure 3:**
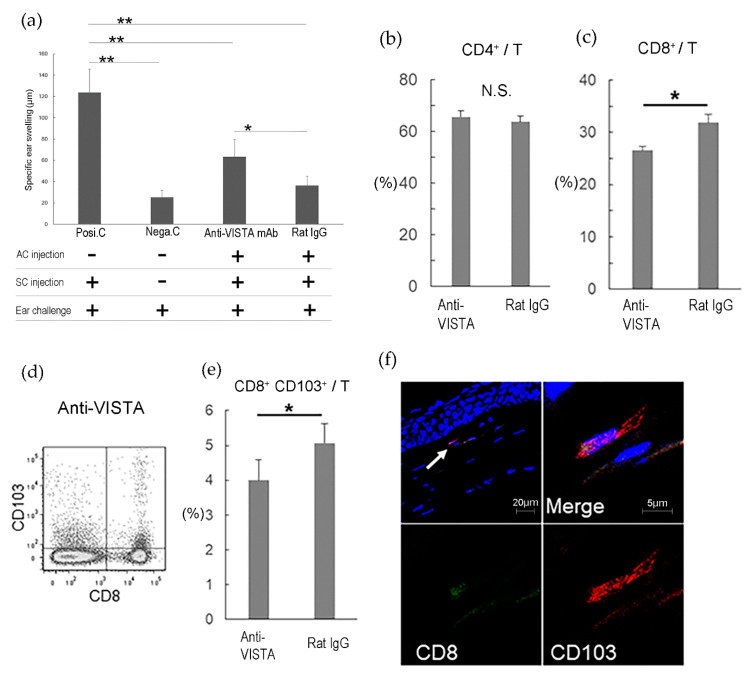
VISTA is involved in the induction of eye-derived tolerance “ACAID” via CD8^+^ CD103^+^ T cells. C57BL/6 spleen cells were used as allo-antigens and injected into the right anterior chamber (AC) of normal eyes of mice treated with anti-VISTA mAb or rat IgG. Two weeks later, C57BL/6 spleen cells were injected subcutaneously to sensitize the mice. After one more week, a challenge was conducted by injecting C57BL/6 spleen cells into the right ear pinna of each mouse, and specific ear swelling was measured 24 h later as an indication of delayed-type hypersensitivity. Positive control mice (Posi.C) received subcutaneous immunization and ear challenge without previous AC injection. Negative control mice (Nega.C) received only the ear challenge without AC injection or immunization. (**a**) shows the results of ACAID induction. (*n* = 6 per group). Data were analyzed using the two-tailed Student’s *t*-test. “SC” denotes subcutaneous. Seventy-two hours after induction of ACAID, expression of CD4, CD8, and CD8/CD103 in T cells from the spleen of ACAID model mice treated with anti-VISTA mAb or rat IgG was examined with flow cytometry. (**b**) shows the proportion of CD4^+^ T cells among T cells. (**c**) shows the proportion of CD8^+^ T cells among T cells. (**d**) shows representative flow cytometry data of CD8^+^ CD103^+^ T cells, and (**e**) shows the proportion of CD8^+^ CD103^+^ T cells among T cells. (**f**) Cryostat sections of surviving corneal allografts at 3 weeks were stained with FITC-conjugated anti-CD8 mAb (green) and PE-conjugated anti-CD103 mAb (red). Nuclei were stained with DAPI (blue). The arrow shows the CD8^+^ CD103^+^ cells, and a double-positive cell at higher magnification is shown. Data are the mean ± SD of three experiments per group. * *p* < 0.05, ** *p* < 0.005, N.S.; not significant. Data were analyzed using the two-tailed Student’s *t*-test. Reprinted from Invest Ophthalmol Vis Sci, 60, 4958–4965. Kunishige T; Taniguchi H; Ohno T; Azuma M; Hori J. VISTA Is Crucial for Corneal Allograft Survival and Maintenance of Immune Privilege. (2019) with permission from IOVS [30].

In the eye, VISTA mRNA is constitutively expressed in the cornea, and the expression of VISTA is localized to CD11b^+^ cells on the corneal stroma [30]. The survival of corneal allograft in the recipients treated with anti-VISTA mAb was less than that of the control. VISTA also involved in the induction of ACAID. ACAID was induced less efficiently in BALB/c mice treated with VISTA mAb. The proportions of CD8^+^ T cells and CD8^+^ CD103^+^ T cells (CD8^+^ T regulatory cells) in the spleen of BALB/c mice treated with anti-VISTA mAb were significantly lower than those of the control. Thus, VISTA plays an essential role in the induction of ACAID via CD8^+^ CD103^+^ T regulatory cells which suppresses T cell infiltration into the cornea [30] (Figure 3).

#### 3.2.3. GITR Ligand and GITR

The pathway between glucocorticoid-induced TNF receptor family-related protein (GITR) and GITR ligand (GITRL) have been shown to control the function of Tregs. GITR is a type I transmembrane protein of the TNF receptor superfamily [62,63]. In the eye, GITRL is expressed constitutively in the cornea and iris–ciliary body [31]. When GITRL was blocked by peritoneal injection of anti-GITRL mAb in recipients of corneal allografts, the allografts became more vulnerable to rejection [31]. This is caused by a GITRL-induced expansion of Foxp3^+^GITR^+^CD25^+^CD4^+^ Tregs within the cornea after corneal transplantation. Depletion of CD4^+^CD25^+^ Tregs also accelerated allograft rejection. In vitro culture system of corneal tissue and T cells, Foxp3^+^CD25^+^CD4^+^ T cells were increased after co-culture with a GITRL-expressing cornea, but not with a GITRL-blocked cornea. Destruction of corneal endothelial cells by T cells was significantly enhanced in GITRL-blocked corneas compared with control corneas. Thus, GITRL-dependent expansion of Foxp3^+^CD4^+^CD25^+^ Tregs within the cornea is one of the mechanisms underlying the immune privilege in corneal allografts [31].

### 3.3. Other Molecules Contributing to Treg in the Cornea

Several in vitro studies have demonstrated that corneal endothelial cells contribute to local immune tolerance in the human eye, as activated T cells exposed to human cultured corneal endothelial cells fail to acquire effector T-cell function [64,65,66]. Cultured human corneal endothelial cells express membrane-bound active TGF-β2 and regulate activation of CD8^+^ T cells via a membrane-bound form of TGF-β [67]. Furthermore, cultured corneal endothelial cells are capable of converting CD8^+^ T cells into Tregs through membrane-bound active TGF-β. Corneal endothelial cell-induced CD8^+^ Tregs expressing CD25^high^ and Foxp3 suppress bystander effector T-cell activation [67]. Encounters between corneal endothelial cells and activated T cells lead to the generation of regulatory T cells. Tregs generated by corneal endothelial cells contribute to the creation of corneal immune privilege via suppression of bystander effector T cells.

Cytotoxic T lymphocyte-associated antigen-2 alpha (CTLA-2α cystein proteinase inhibitor) expressed on murine corneal endothelial cells, contributes to the corneal endothelial cell-dependent suppression of bystander T-cell activation and the generation of CD4^+^ Tregs through TGF-β production [18].

## 4. Dry Eye as irAE Induced by Immune Checkpoint Inhibitors

### 4.1. Immune-Related Adverse Events (irAEs)

Certain tumors have immune privilege, and express immune checkpoint molecules to escape from the immune system. Antibodies targeting the immune-checkpoint proteins CTLA-4, PD-1, and PD-L have become new therapies for cancer [68,69,70,71,72,73]. These immune checkpoint inhibitors enhance the immune system by releasing inhibition on T cells, and cause auto-immune/auto-inflammatory side effects called “immune-related adverse events (irAEs)”. The most common irAEs are noted in skin (rash), gastrointestinal tract (colitis, hepatitis, pancreatitis), lung (pneumonitis), heart (myocarditis), and endocrine system (thyroiditis, hypophysitis) [74]. Rheumatic irAEs such as inflammatory arthritis, polymyalgia-like syndromes, myosis, sicca syndrome, sarcoidosis, and vasculitis are also common and develop in ~5–10% of patients treated with immune checkpoint inhibitors [75]. Immune checkpoint inhibitors abolish not only self-tolerance but also immune privilege in the privileged organs such as in the eye, ocular inflammation is induced.

**Figure 4:**
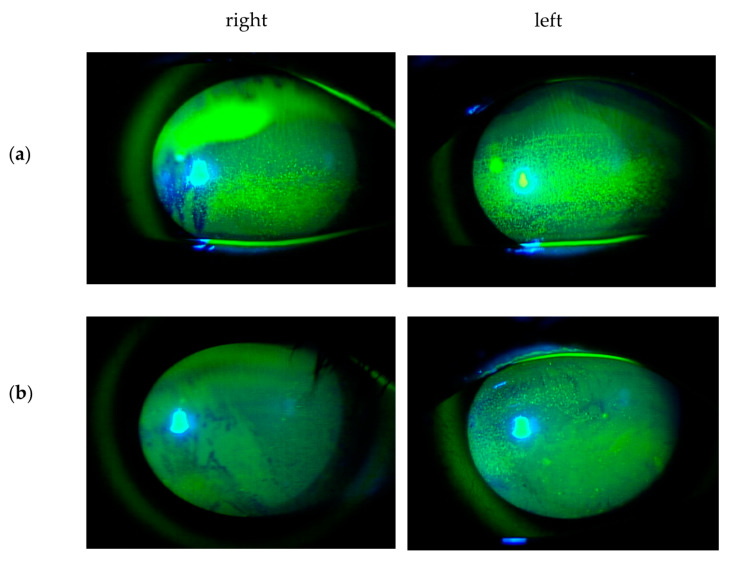
Slit-lamp images of the corneal surface with fluorescein staining in dry eye induced by Pembrolizumab (anti-PD1 antibody). A 44-year old man with metastatic lung cancer from a primary kidney cancer was referred to the ocular inflammation service, Nippon Medical School Hospital, for bilateral red eyes. he was undergoing his second cycle of Pembrolizumab (anti-PD1 antibody). (**a**) He had bilateral punctate epithelial erosions with moderate fluorescein staining. The basic tear secretion test was 0 mm in the right eye and 3 mm in the left eye. His examination revealed Vogt–Koyanagi–Harada (VKH)-like syndrome, hepatic dysfunction, hypopituitarism, and adrenal dysfunction as irAEs. Pembrolizumab was discontinued. He was administered oral hydrocortisone (25 mg/day) for his adrenal dysfunction. At 12 weeks after topical artificial tears and topical rebamipide (2%), his dry eye gradually improved and showed (**b**) only minimal fluorescein staining of punctate epithelial erosions with the basic tear secretion test 20 mm in the right eye and 15 mm in the left eye.

Ophthalmic irAEs of immune checkpoint inhibitors most frequently manifest as uveitis such as Vogt–Koyanagi–Harada disease (VKH) syndrome, and dry eye (Figure 4). Myasthenia gravis, inflammatory orbitopathy, keratitis, cranial nerve palsy, optic neuropathy, serous retinal detachment, extraocular muscle myopathy, atypical chorioretinal lesions, immune retinopathy, and neuroretinitis have also reported as ophthalmic irAEs [76]. Mild irAEs can be treated with topical or periocular corticosteroids, whereas systemic corticosteroids and discontinuation of checkpoint inhibitors are indicated in severe inflammation [77].

### 4.2. Immunopathologic Mechanisms of Dry Eye Associated with Checkpoint Inhibitor Therapy

Dry eye is common irAE and develop in ~1–24% of patients treated with immune checkpoint inhibitors [76]. There are three hypothesized immunopathologic mechanisms of dry eye associated with immune checkpoint inhibitors. The first mechanism is that immune checkpoint inhibitors abolish self-tolerance and induce autoimmunity that lead to Rheumatic Sicca syndrome with lacrimal grand dysfunction [75]. The second one is that immune checkpoint inhibitors induce T cell infiltration in the ocular surface. It has been reported that dry eye associated with Nivolmab (anti-PD-1 antibody) progressed to corneal perforation, and that topical cyclosporine was an effective treatment [78]. It is suggested that dry eye induced by anti-PD-1 antibody is medicated by T cell, because cyclosporine downregulates T cell activity [78]. As described in the above Section 3, PD-1 is crucial for corneal immune privilege by inducing T cell apoptosis in the cornea. Blockade of PD-1 can abolish corneal immune privilege and allow T cell Infiltration in the ocular surface. The last one is that immune checkpoint inhibitors induce sarcoidosis-like granulomatous inflammation of lacrimal grands, as reported in [79]. Sarcoidosis has been reported in patients with cancer treated with ipilimumab (anti-CTLA4 antibody) [80,81]. It is suggested that granulomatous infiltration induced by immune checkpoint inhibitors is attributed to lymphocytic infiltrate with CD8+ T cells and IL-2 secretion by activated T cells is thought to play a role in the pathogenesis of sarcoid-like granulomatous [82].

## 5. Conclusions

The eye, which is endowed with immune privilege, is a rare organ that permits analysis of the self-regulatory mechanisms for inflammation in organs. As described above, immune checkpoints are constitutively expressed in the cornea, and regulate immune responses in order to prevent inflammation-mediated corneal tissue damage. Immune checkpoints inhibitors have achieved remarkable survival benefits as cancer therapies; however, their responses resulted in a broad spectrum of irAE in various organs and dry eye is no exception. Understanding how immune privilege can be modified in corneal inflammation including dry eye will lead to the development of new therapeutic approaches to other ocular inflammatory diseases, tissue transplantations, and autoimmune diseases.

## Figures and Tables

**Table 1:** Immunomodulatory factors expressed in the anterior segment of the eye. Reprinted from Prog Retin Eye Res. 72, 100758. Hori, J.; Yamaguchi, T.; Keino, H.; Hamrah, P.; Maruyama, K. Immune privilege in corneal transplantation. Copyright (2019), with permission from Elsevier [3].

Soluble Factors in the Anterior Chamber (Target Cells/Factors to Suppress)	Cell Surface Molecules of the Cornea and Iris–Ciliary Body (Target Cells/Factors to Suppress)
α-MSH (T cells, macrophages, neutrophils)	PD-L1 (B7-H1) (T cells)
VIP (T cells)	ICOSL (B7-H2, B7RP-1) (T cells)
Somatostatin (T cells)	VISTA (APCs, T cells)
CGRP (macrophages)	GITR ligand (T cells)
TGF-β2 (T cells, macrophages, NK cells)	Galectin-9 (T cells)
TSP-1 (macrophages)	TRAIL (T cells)
MIF (NK cells)	B7-2 (via CTLA4) (T cells)
IL-1Ra (IL-1)	CTLA-2α (T cells)
sFas L (T cells, neutrophils)	Fas L (CD95 L) (T cells, neutrophils)
CD46, CD55, CD59, C3ib (complement)	MHC class Ib (T cells, NK cells)
	CD46, CD55, CD59 (complement)

α-MSH: alpha-Melanocyte stimulating hormone, VIP: vasoactive intestinal peptide, CGRP: calcitonin gene-related peptide, TGF-β2: transforming growth factor-beta 2, TSP-1: thrombospondin-1, MIF: macrophage migrating inhibitory factor, IL-1Ra: interleukin 1 receptor antagonist, sFas L: soluble Fas ligand, PD-L1: programmed death ligand-1, ICOSL: inducible costimulatory molecule ligand, VISTA: V-domain Ig suppressor of T cell activation, GITR: glucocorticoid-induced tumor necrosis factor receptor family-related protein, TRAIL: tumor necrosis factor related apoptosis-inducing ligand, CTLA4: cytotoxic T lymphocyte-associated antigen 4, CTLA-2α: cytotoxic T lymphocyte-associated antigen-2 alpha.

## Abbreviations

ACAID	anterior chamber-associated immune deviation
α-MSH	alpha-Melanocyte stimulating hormone
VIP	vasoactive intestinal peptide
CGRP	calcitonin gene-related peptide
TGF-β2	transforming growth factor-beta 2
TSP-1	thrombospondin-1
MIF	macrophage migrating inhibitory factor
IL-1Ra	interleukin 1 receptor antagonist
sFas L	soluble Fas ligand
PD-1	programmed death-1
PD-L1	programmed death ligand-1
Gal-9	Galectin-9
Tim-3	T-cell immunoglobulin and mucin domain-3
ICOS	inducible costimulatory molecule
ICOSL	inducible costimulatory molecule ligand
VISTA	V-domain Ig suppressor of T cell activation
GITR	glucocorticoid-induced tumor necrosis factor receptor family-related protein
GITRL	glucocorticoid-induced tumor necrosis factor receptor family-related protein ligand
TRAIL	tumor necrosis factor related apoptosis-inducing ligand
CTLA4	cytotoxic T lymphocyte-associated antigen 4
CTLA-2α	cytotoxic T lymphocyte-associated antigen-2 alpha
APCs	antigen presenting cells
mAb	monoclonal antibody
Treg	regulatory T cells
irAE	immune-related adverse event

## References

[1] Niederkorn J.Y. (2006). See no evil, hear no evil, do no evil: The lessons of immune privilege. Nat. Immunol..

[2] Streilein J.W. (2003). Ocular immune privilege: Therapeutic opportunities from an experiment of nature. Nat. Rev. Immunol..

[3] Hori J., Yamaguchi T., Keino H., Hamrah P., Maruyama K. (2019). Immune privilege in corneal transplantation. Prog. Retin. Eye Res..

[4] Medawar P.B. (1948). Immunity to homologous grafted skin; the fate of skin homografts transplanted to the brain, to subcutaneous tissue, and to the anterior chamber of the eye. Br. J. Exp. Pathol..

[5] Barker C.F., Billingham R.E. (1977). Immunologically privileged sites. Adv. Immunol..

[6] Streilein J.W. (1987). Immune regulation and the eye: A dangerous compromise. FASEB J..

[7] Niederkorn J.Y. (1990). Immune privilege and immune regulation in the eye. Adv. Immunol..

[8] Forrester J.V. (2009). Privilege revisited: An evaluation of the eye’s defence mechanism. Eye.

[9] Pleyer U., Schlickeiser S. (2009). The taming of the shrew? The immunology of corneal transplantation. Acta Ophthalmol..

[10] Apte R.S., Sinha D., Mayhew E., Wistow G.J., Niederkorn J.Y. (1998). Cutting edge: Role of macrophage migration inhibitory factor in inhibiting NK cell activity and preserving immune privilege. J. Immunol..

[11] Griffith T.S., Brunner T., Fletcher S.M., Green D.R., Ferguson T.A. (1995). Fas ligand-induced apoptosis as a mechanism of immune privilege. Science.

[12] Kennedy M.C., Rosenbaum J.T., Brown J., Planck S.R., Huang X., Armstrong C.A., Ansel J.C. (1995). Novel production of interleukin-1 receptor antagonist peptides in normal human cornea. J. Clin. Investig..

[13] Namba K., Kitaichi N., Nishida T., Taylor A.W. (2002). Induction of regulatory T cells by the immunomodulating cytokines alpha-melanocyte-stimulating hormone and transforming growth factor-beta2. J. Leukoc. Biol..

[14] Sheibani N., Sorenson C.M., Cornelius L.A., Frazier W.A. (2000). Thrombospondin-1, a natural inhibitor of angiogenesis, is present in vitreous and aqueous humor and is modulated by hyperglycemia. Biochem. Biophys. Res. Commun..

[15] Sohn J.H., Kaplan H.J., Suk H.J., Bora P.S., Bora N.S. (2000). Complement regulatory activity of normal human intraocular fluid is mediated by MCP, DAF, and CD59. Investig. Ophthalmol. Vis. Sci..

[16] Stuart P.M., Griffith T.S., Usui N., Pepose J., Yu X., Ferguson T.A. (1997). CD95 ligand (FasL)-induced apoptosis is necessary for corneal allograft survival. J. Clin. Investig..

[17] Sugita S., Streilein J.W. (2003). Iris pigment epithelium expressing CD86 (B7-2) directly suppresses T cell activation in vitro via binding to cytotoxic T lymphocyte-associated antigen 4. J. Exp. Med..

[18] Sugita S., Yamada Y., Horie S., Nakamura O., Ishidoh K., Yamamoto Y., Yamagami S., Mochizuki M. (2011). Induction of T regulatory cells by cytotoxic T-lymphocyte antigen-2alpha on corneal endothelial cells. Investig. Ophthalmol. Vis. Sci..

[19] Taylor A.W., Yee D.G., Streilein J.W. (1998). Suppression of nitric oxide generated by inflammatory macrophages by calcitonin gene-related peptide in aqueous humor. Investig. Ophthalmol. Vis. Sci..

[20] Wilbanks G.A., Mammolenti M., Streilein J.W. (1992). Studies on the induction of anterior chamber-associated immune deviation (ACAID). III. Induction of ACAID depends upon intraocular transforming growth factor-beta. Eur. J. Immunol..

[21] Yamagami S., Kawashima H., Tsuru T., Yamagami H., Kayagaki N., Yagita H., Okumura K., Gregerson D.S. (1997). Role of Fas-Fas ligand interactions in the immunorejection of allogeneic mouse corneal transplants. Transplantation.

[22] Niederkorn J.Y. (2007). The induction of anterior chamber-associated immune deviation. Chem. Immunol. Allergy.

[23] Stein-Streilein J., Streilein J.W. (2002). Anterior chamber associated immune deviation (ACAID): Regulation, biological relevance, and implications for therapy. Int. Rev. Immunol..

[24] Kaplan H.J., Streilein J.W. (1977). Immune response to immunization via the anterior chamber of the eye. I. F. lymphocyte-induced immune deviation. J. Immunol..

[25] Kaplan H.J., Streilein J.W. (1978). Immune response to immunization via the anterior chamber of the eye. II. An analysis of F1 lymphocyte-induced immune deviation. J. Immunol..

[26] Ksander B.R., Streilein J.W. (1989). Analysis of cytotoxic T cell responses to intracameral allogeneic tumors. Investig. Ophthalmol. Vis. Sci..

[27] Wilbanks G.A., Streilein J.W. (1990). Distinctive humoral immune responses following anterior chamber and intravenous administration of soluble antigen. Evidence for active suppression of IgG2-secreting B lymphocytes. Immunology.

[28] Hori J., Wang M., Miyashita M., Tanemoto K., Takahashi H., Takemori T., Okumura K., Yagita H., Azuma M. (2006). B7-H1-induced apoptosis as a mechanism of immune privilege of corneal allografts. J. Immunol..

[29] Kunishige T., Taniguchi H., Terada M., Akiba H., Yagita H., Abe R., Hori J. (2016). Protective Role of ICOS and ICOS Ligand in Corneal Transplantation and in Maintenance of Immune Privilege. Investig. Ophthalmol. Vis. Sci..

[30] Kunishige T., Taniguchi H., Ohno T., Azuma M., Hori J. (2019). VISTA Is Crucial for Corneal Allograft Survival and Maintenance of Immune Privilege. Investig. Ophthalmol. Vis. Sci..

[31] Hori J., Taniguchi H., Wang M., Oshima M., Azuma M. (2010). GITR ligand-mediated local expansion of regulatory T cells and immune privilege of corneal allografts. Investig. Ophthal. Vis. Sci..

[32] Shimmura-Tomita M., Wang M., Taniguchi H., Akiba H., Yagita H., Hori J. (2013). Galectin-9-mediated protection from allo-specific T cells as a mechanism of immune privilege of corneal allografts. PLoS ONE.

[33] Nagata S., Golstein P. (1995). The Fas death factor. Science.

[34] Ashkenazi A., Dixit V.M. (1998). Death receptors: Signaling and modulation. Science.

[35] Ashkenazi A., Dixit V.M. (1999). Apoptosis control by death and decoy receptors. Curr. Opin. Cell Biol..

[36] Wolf B.B., Green D.R. (1999). Suicidal tendencies: Apoptotic cell death by caspase family proteinases. J. Biol. Chem..

[37] Nagata S. (1997). Apoptosis by death factor. Cell.

[38] Hori J., Joyce N., Streilein J.W. (2000). Epithelium-deficient corneal allografts display immune privilege beneath the kidney capsule. Investig. Ophthalmol. Vis. Sci..

[39] Hori J., Joyce N.C., Streilein J.W. (2000). Immune privilege and immunogenicity reside among different layers of the mouse cornea. Investig. Ophthalmol. Vis. Sci..

[40] Ishida Y., Agata Y., Shibahara K., Honjo T. (1992). Induced expression of PD-1, a novel member of the immunoglobulin gene superfamily, upon programmed cell death. EMBO J..

[41] Nishimura H., Nose M., Hirai H., Minato N., Honjo T. (1999). Development of lupus-like autoimmune diseases by disruption of the PD-1 gene encoding and ITIM motif-carrying immunoreceptor. Immunity.

[42] Sharpe A.H., Freeman G.J. (2002). The B7-CD28 superfamily. Nat. Rev. Immunol..

[43] Dong H., Zhu G., Tamada K., Chen L. (1999). B7-H1, a third member of the B7 family, co-stimulates T-cell proliferation and interleukin-10 secretion. Nat. Med..

[44] Dong H., Strome S.E., Salomao D.R., Tamura H., Hirano F., Flies D.B., Roche P.C., Lu J., Zhu G., Tamada K. (2002). Tumor-associated B7-H1 promotes T-cell apoptosis: A potential mechanism of immune evasion. Nat. Med..

[45] Dong H., Zhu G., Tamada K., Flies D.B., van Deursen J.M., Chen L. (2004). B7-H1 determines accumulation and deletion of intrahepatic CD8(+) T lymphocytes. Immunity.

[46] Rodriguez-Manzanet R., DeKruyff R., Kuchroo V.K., Umetsu D.T. (2009). The costimulatory role of TIM molecules. Immunol. Rev..

[47] Ueno T., Habicht A., Clarkson M.R., Albin M.J., Yamaura K., Boenisch O., Popoola J., Wang Y., Yagita H., Akiba H. (2008). The emerging role of T cell Ig mucin 1 in alloimmune responses in an experimental mouse transplant model. J. Clin. Investig..

[48] Xiao S., Najafian N., Reddy J., Albin M., Zhu C., Jensen E., Imitola J., Korn T., Anderson A.C., Zhang Z. (2007). Differential engagement of Tim-1 during activation can positively or negatively costimulate T cell expansion and effector function. J. Exp. Med..

[49] Monney L., Sabatos C.A., Gaglia J.L., Ryu A., Waldner H., Chernova T., Manning S., Greenfield E.A., Coyle A.J., Sobel R.A. (2002). Th1-specific cell surface protein Tim-3 regulates macrophage activation and severity of an autoimmune disease. Nature.

[50] Sanchez-Fueyo A., Tian J., Picarella D., Domenig C., Zheng X.X., Sabatos C.A., Manlongat N., Bender O., Kamradt T., Kuchroo V.K. (2003). Tim-3 inhibits T helper type 1-mediated auto- and alloimmune responses and promotes immunological tolerance. Nat. Immunol..

[51] Sabatos C.A., Chakravarti S., Cha E., Schubart A., Sanchez-Fueyo A., Zheng X.X., Coyle A.J., Strom T.B., Freeman G.J., Kuchroo V.K. (2003). Interaction of Tim-3 and Tim-3 ligand regulates T helper type 1 responses and induction of peripheral tolerance. Nat. Immunol..

[52] Anderson A.C., Anderson D.E., Bregoli L., Hastings W.D., Kassam N., Lei C., Chandwaskar R., Karman J., Su E.W., Hirashima M. (2007). Promotion of tissue inflammation by the immune receptor Tim-3 expressed on innate immune cells. Science.

[53] Zhu C., Anderson A.C., Schubart A., Xiong H., Imitola J., Khoury S.J., Zheng X.X., Strom T.B., Kuchroo V.K. (2005). The Tim-3 ligand galectin-9 negatively regulates T helper type 1 immunity. Nat. Immunol..

[54] Hutloff A., Dittrich A.M., Beier K.C., Eljaschewitsch B., Kraft R., Anagnostopoulos I., Kroczek R.A. (1999). ICOS is an inducible T-cell co-stimulator structurally and functionally related to CD28. Nature.

[55] Yoshinaga S.K., Whoriskey J.S., Khare S.D., Sarmiento U., Guo J., Horan T., Shih G., Zhang M., Coccia M.A., Kohno T. (1999). T-cell co-stimulation through B7RP-1 and ICOS. Nature.

[56] Tezuka K., Tsuji T., Hirano D., Tamatani T., Sakamaki K., Kobayashi Y., Kamada M., Tafuri-Bladt A. (2000). Identification and characterization of rat AILIM/ICOS, a novel T-cell costimulatory molecule, related to the CD28/CTLA4 family. Biochem. Biophys. Res. Commun..

[57] Swallow M.M., Wallin J.J., Sha W.C. (1999). B7h, a novel costimulatory homolog of B7.1 and B7.2, is induced by TNFalpha. Immunity.

[58] Wang L., Rubinstein R., Lines J.L., Wasiuk A., Ahonen C., Guo Y., Lu L.-F., Gondek D., Wang Y., Fava R.A. (2011). VISTA, a novel mouse Ig superfamily ligand that negatively regulates T cell responses. J. Exp. Med..

[59] Flies D.B., Wang S., Xu H., Chen L. (2011). Cutting edge: A monoclonal antibody specific for the programmed death-1 homolog prevents graft-versus-host disease in mouse models. J. Immunol..

[60] Flies D.B., Han X., Higuchi T., Zheng L., Sun J., Ye J.J., Chen L. (2014). Coinhibitory receptor PD-1H preferentially suppresses CD4(+) T cell-mediated immunity. J. Clin. Investig..

[61] Deng J., Le Mercier I., Kuta A., Noelle R.J. (2016). A New VISTA on combination therapy for negative checkpoint regulator blockade. J. Immunother. Cancer.

[62] Gurney A.L., Marsters S.A., Huang R.M., Pitti R.M., Mark D.T., Baldwin D.T., Gray A.M., Dowd A.D., Brush A.D., Heldens A.D. (1999). Identification of a new member of the tumor necrosis factor family and its receptor, a human ortholog of mouse GITR. Curr. Biol..

[63] Nocentini G., Giunchi L., Ronchetti S., Krausz L.T., Bartoli A., Moraca R., Migliorati G., Riccardi C. (1997). A new member of the tumor necrosis factor/nerve growth factor receptor family inhibits T cell receptor-induced apoptosis. Proc. Natl. Acad. Sci. USA.

[64] Kawashima H., Prasad S.A., Gregerson D.S. (1994). Corneal endothelial cells inhibit T cell proliferation by blocking IL-2 production. J. Immunol..

[65] Obritsch W.F., Kawashima H., Evangelista A., Ketcham J.M., Holland E.J., Gregerson D.S. (1992). Inhibition of in vitro T cell activation by corneal endothelial cells. Cell. Immunol..

[66] Sugita S., Usui Y., Horie S., Futagami Y., Yamada Y., Ma J., Kezuka T., Hamada H., Usui T., Mochizuki M. (2009). Human corneal endothelial cells expressing programmed death-ligand 1 (PD-L1) suppress PD-1^+^ T helper 1 cells by a contact-dependent mechanism. Investig. Ophthalmol. Vis. Sci..

[67] Yamada Y., Sugita S., Horie S., Yamagami S., Mochizuki M. (2010). Mechanisms of immune suppression for CD8+ T cells by human corneal endothelial cells via membrane-bound TGFbeta. Investig. Ophthalmol. Vis. Sci..

[68] Zou W., Chen L. (2008). Inhibitory B7-family molecules in the tumour microenvironment. Nat. Rev. Immunol..

[69] Pardoll D., Drake C. (2012). Immunotherapy earns its spot in the ranks of cancer therapy. J. Exp. Med..

[70] Sharma P., Allison J.P. (2015). The future of immune checkpoint therapy. Science.

[71] Postow M.A., Callahan M.K., Wolchok J.D. (2015). Immune Checkpoint Blockade in Cancer Therapy. J. Clin. Oncol..

[72] Sharma P., Allison J.P. (2015). Immune checkpoint targeting in cancer therapy: Toward combination strategies with curative potential. Cell.

[73] Xu W., Hieu T., Malarkannan S., Wang L. (2018). The structure, expression, and multifaceted role of immune-checkpoint protein VISTA as a critical regulator of anti-tumor immunity, autoimmunity, and inflammation. Cell. Mol. Immunol..

[74] Baxi S., Yang A., Gennarelli R.L., Khan N., Wang Z., Boyce L., Korenstein D. (2018). Immune-related adverse events for anti-PD-1 and anti-PD-L1 drugs: Systematic review and meta-analysis. BMJ.

[75] Abdel-Wahab N., Suarez-Almazor M.E. (2019). Frequency and distribution of various rheumatic disorders associated with checkpoint inhibitor therapy. Rheumatology.

[76] Dalvin L.A., Shields C.L., Orloff M., Sato T., Shields J.A. (2018). CHECKPOINT INHIBITOR IMMUNE THERAPY: Systemic Indications and Ophthalmic Side Effects. Retina.

[77] Antoun J., Titah C., Cochereau I. (2016). Ocular and orbital side-effects of checkpoint inhibitors: A review article. Curr. Opin. Oncol..

[78] Nguyen A.T., Elia M., Materin M.A., Sznol M., Chow J. (2016). Cyclosporine for Dry Eye Associated with Nivolumab: A Case Progressing to Corneal Perforation. Cornea.

[79] Ileana Dumbrava E., Smith V., Alfattal R., El-Naggar A.K., Penas-Prado M., Tsimberidou A.M. (2018). Autoimmune Granulomatous Inflammation of Lacrimal Glands and Axonal Neuritis Following Treatment with Ipilimumab and Radiation Therapy. J. Immunother..

[80] Eckert A., Schoeffler A., Dalle S., Phan A., Kiakouama L., Thomas L. (2008). Anti-CTLA4 monoclonal antibody induced sarcoidosis in a metastatic melanoma patient. Dermatology.

[81] Suozzi K.C., Stahl M., Ko C.J., Chiang A., Gettinger S.N., Siegel M.D., Bunick C.G. (2016). Immune-related sarcoidosis observed in combination ipilimumab and nivolumab therapy. JAAD Case Rep..

[82] Ziegenhagen M.W., Müller-Quernheim J. (2003). The cytokine network in sarcoidosis and its clinical relevance. J. Intern. Med..

